# Calsyntenins Are Expressed in a Dynamic and Partially Overlapping Manner during Neural Development

**DOI:** 10.3389/fnana.2017.00076

**Published:** 2017-08-30

**Authors:** Gemma de Ramon Francàs, Tobias Alther, Esther T. Stoeckli

**Affiliations:** Department of Molecular Life Sciences and Neuroscience Center Zurich, University of Zurich Zurich, Switzerland

**Keywords:** spinal cord development, axon guidance, chicken embryo, neural circuit formation, calsyntenins, cerebellar development, tectum mesencephali, retinal development

## Abstract

Calsyntenins form a family of linker proteins between distinct populations of vesicles and kinesin motors for axonal transport. They were implicated in synapse formation and synaptic plasticity by findings in worms, mice and humans. These findings were in accordance with the postsynaptic localization of the Calsyntenins in the adult brain. However, they also affect the formation of neural circuits, as loss of Calsyntenin-1 (Clstn1) was shown to interfere with axonal branching and axon guidance. Despite the fact that Calsyntenins were discovered originally in embryonic chicken motoneurons, their distribution in the developing nervous system has not been analyzed in detail so far. Here, we summarize our analysis of the temporal and spatial expression patterns of the cargo-docking proteins Clstn1, Clstn2 and Clstn3 during neural development by comparing the dynamic distribution of their mRNAs by *in situ* hybridization in the spinal cord, the cerebellum, the retina and the tectum, as well as in the dorsal root ganglia (DRG).

## Introduction

Delivery of proteins to specific cellular destinations is crucial for neural circuit formation and synaptic plasticity. Therefore, the transport of vesicular cargo has to be regulated precisely both temporally and spatially. Calsyntenins, a family of three transmembrane proteins, have been identified as cargo-docking proteins in vesicular transport along axons (Vogt et al., 2001; Hintsch et al., 2002; Konecna et al., 2006). Mutations in the two kinesin-binding domains of Calsyntenin-1 (Clstn1) significantly reduced fast anterograde axonal transport of vesicles (Konecna et al., 2006), and interfered with the delivery of specific cargo to growth cones and synapses. The importance of tight regulation of trafficking was demonstrated during several distinct steps of neural circuit formation. Clstn1 was shown to regulate axon branching (Ponomareva et al., 2014; Lee et al., 2017) and axon guidance at choice points (Alther et al., 2016). All calsyntenins were shown to affect synaptogenesis and synaptic plasticity (Pettem et al., 2013; Um et al., 2014). For example, juvenile Clstn1 knockout mice exhibited enhanced long-term potentiation (LTP), in agreement with the observed effect on spine morphology (Ster et al., 2014). Changes in cognitive functions have been associated with calsyntenins in health and disease. Specific polymorphisms in the calsyntenin-2 (Clstn2) gene were linked to enhanced episodic memory in healthy humans (Preuschhof et al., 2010). Changes in cognitive abilities in the absence of Clstn2 were observed in mice (Lipina et al., 2016) and worms (Ikeda et al., 2008; Hoerndli et al., 2009).

A link between calsyntenin and memory was also provided by studies addressing the differences between normal aged brains and brains from patients diagnosed with Alzheimer’s disease (Ringman et al., 2012; Vagnoni et al., 2012). These findings are interesting in the context of cell biological and biochemical analyses that link Calsyntenins to neuronal APP transport (Araki et al., 2003; Ludwig et al., 2009; Steuble et al., 2010, 2012). By sheltering APP from cleavage by the α-secretase ADAM10, Clstn1 was suggested to contribute to the transport of full-length APP to the cell surface. In its absence Aβ-production was increased (Steuble et al., 2012; Vagnoni et al., 2012).

Northern blot analysis localized Clstn2 and calsyntenin-3 (Clstn3) mRNAs exclusively to brain tissue in adult mice. Clstn1 was also found predominantly in the brain, but much lower levels were also detected in non-neuronal tissues, such as kidney, lung and heart. A more detailed analysis in the adult mouse brain revealed calsyntenin expression in most brain regions (Hintsch et al., 2002). Clstn1 was found at high levels in most neurons. In contrast, high Clstn2 levels were only found in a subpopulation of neurons but still in many different brain areas. Clstn3 expression often resembled Clstn1 with respect to distribution in different brain areas, but it was more similar to Clstn2 than Clstn1 with respect to variability of expression levels between different subpopulations of cells (Hintsch et al., 2002). In the developing brain, Clstn1 was found enriched in fiber tracts associated with tubulovesicular organelles (Konecna et al., 2006). In the adult mouse, Clstn1 protein was only found at very low levels in fiber tracts. At the electron microscopic level, Clstn1 was found in the post-synaptic membrane and in the spine apparatus of dendritic spines. The distinct localization of Clstn1 fragments is consistent with proteolytic cleavage of full-length Calsyntenin in the synaptic cleft resulting in the release of the N-terminal fragment and followed by the internalization of the transmembrane stump into the spine apparatus (Vogt et al., 2001). The cleavage site of Clstn1 is strongly conserved in all three Calsyntenins and overall human, murine and chicken Calsyntenins are highly conserved (Hintsch et al., 2002).

Clstn1 was initially identified in motoneuron cultures derived from embryonic chicken spinal cords (Vogt et al., 2001), but for its functional analysis studies have largely focused on axonal transport *in vitro* (Konecna et al., 2006; Ludwig et al., 2009; Steuble et al., 2010) or on its role during synaptic plasticity in health (Pettem et al., 2013; Ster et al., 2014; Um et al., 2014) and disease (Steuble et al., 2012; Vagnoni et al., 2012). In contrast to the adult nervous system, very little is known about the expression patterns of the calsyntenin family members during neural development. Therefore, we compare the temporal and spatial expression pattern of all three calsyntenins in selected neuronal populations of the developing central and peripheral nervous system in the chicken embryo.

## Materials and Methods

### Tissue Preparation

Fertilized eggs were incubated at 39°C and 55%–65% humidity. Embryos were staged according to Hamburger and Hamilton (1951). Embryos were sacrificed and dissected in cold phosphate-buffered saline (PBS, pH 7.4) and fixed in 4% paraformaldehyde at room temperature for different times depending on the stage (see Table 1). To make cutting easier, old spinal cords (HH38 and HH44) were treated with Morse’s solution (10% sodium citrate, 22.5% formic acid; Shibata et al., 2000) for 12–36 h, respectively. Embryos were cryoprotected in 25% sucrose, and then embedded in Tissue-Tek O.C.T Compound (Sakura). Specimens were frozen in isopentane on dry ice and stored at −20°C. Sections of 25 μm thickness were obtained using a cryostat (LEICA CM1850; except for HH12, where we used 30 μm).

**Table 1:** Age-dependent treatment of tissue sections for *in situ* hybridization.

	PFA	Morse’s solution	Proteinase K	Post fixation
**Spinal cord**				
HH12	25	-	-	4
HH18	30	-	-	-
HH24	45	-	-	-
HH30	120	-	-	-
HH34	180	-	-	-
HH38	200	O/N	5	15
HH44	240	O/N	5	15
**Brains**				
HH34	120	-	4	10
HH36	160	-	5	15
HH38	180	-	5	15
HH44	200	-	5	15
**Retina**				
HH34	120	-	4	10
HH38	180	-	5	15
HH44	200	-	5	15

*All times are given in minutes, except for O/N (overnight)*.

This study was carried out in accordance with the recommendations of the national authorities of Switzerland (Animal Protection Ordinance). The protocol and the experiments were approved by the cantonal veterinary office of the Canton Zurich (Kantonales Veterinäramt).

### Probe Preparation for *In Situ* Hybridization

Plasmids containing calsyntenin cDNA fragments (ESTs obtained from Source BioScience) were linearized by digestion with the appropriate restriction enzymes to produce templates for the synthesis of antisense and sense probes. The ESTs used were: ChEST846m5 for Clstn1, ChEST1002c5 for Clstn2 and ChEST882h15 for Clstn3. For linearization, 10 μg plasmid DNA were incubated with 20 U of the restriction enzyme in the appropriate buffer for 2–4 h at 37°C. After phenol/chloroform extraction and acetate/ethanol precipitation, DIG-labeled sense and anti-sense probes were synthesized by *in vitro* transcription. Two microgram linearized plasmid DNA, 2 μl of 10× concentrated DIG RNA Labeling Mix (Roche), 2 μl 100 mM DTT (Promega), 4 μl 5× concentrated transcription buffer (Promega), 1 μl RNasin (40 U/μl; Promega), 2 μl of T3 or T7 RNA polymerase (Roche) and diethyl pyrocarbonate (DEPC)-treated H_2_O were mixed to a final volume of 20 μl and incubated at 37°C for 2 h. The DIG-labeled RNA probes were extracted by lithium chloride precipitation and dissolved in 100 μl DEPC-treated H_2_O.

### *In Situ* Hybridization

For all steps before and including hybridization, DEPC-treated H_2_O and stock solutions were used. Sections taken from embryos older than HH34 were washed twice in PBS for 10 min each and treated with proteinase K (Roche, 1 μg/ml) for 5 min. Sections were rinsed in PBS and post-fixed using 2% PFA for 10–15 min (depending on the stage, see Table 1). Before pre-hybridization, all sections were rinsed twice in PBS and once in H_2_O for 5 min each. Then the sections were acetylated for 10 min in 1% tri-ethanolamine to which 0.25% (vol/vol) acetic anhydride was added with constant stirring. Following two washes in PBS (5 min each) and a wash in 2× SSC (0.3 M NaCl, 0.03 M tri-sodium citrate, pH 7.0) for 5 min, the prehybridization was performed at 56°C for 180–240 min. The prehybridization solution (750 μl per slide) was composed of 50% formamide, 5× SSC, 5× Denhardt’s Solution, 250 μg/ml yeast tRNA (Roche) and 500 μg/ml salmon sperm DNA (Sigma). For hybridization, 500 ng/ml of each RNA probe were added to the prehybridization solution (700 μl per slide) and preheated to 56°C. The hybridization was overnight at 56°C. Both prehybridization and hybridization were performed in a chamber containing paper towels soaked in 50% formamide/5× SSC, properly closed and wrapped with parafilm to avoid evaporation of the hybridization solution.

After hybridization, the sections were washed by dipping the slides in 5× SSC at 56°C, followed by washes of 5 min at 56°C in decreasing concentrations of SSC (5×, 2× and 0.2× SSC). Slices were incubated for 20 min in 50% formamide/0.2× SSC at 56°C and then for 5 min in 0.2× SSC at RT. Then, the sections were rinsed two times in detection buffer (0.1 M Tris-base, 0.15 M NaCl, pH 7.5) for 5–10 min each at RT. Unspecific antibody binding was blocked by incubation in 3% milk powder in detection buffer (blocking buffer) for 120–180 min. Incubation with the anti-DIG-AP antibody (Roche; diluted 1:5000 in blocking buffer; 500 μl per slide) was also for 120–180 min. Unbound antibody was rinsed off by two 15-min washes in detection buffer. Hybridization was visualized by incubation in AP buffer (337.5 μg/ml nitroblue tetrazolium (NBT; Roche), 175 μg/ml 5-bromo-4-chloro-3-indoyl phosphate (BCIP; Roche) and 240 μg/ml levamisole (Sigma) in 0.1 M Tris-base, 0.1 M NaCl, 50 mM MgCl_2_, pH 9.5; 700 μl per slide) for 15–16 h in a dark humidified chamber. The reaction was stopped by dipping the sections in TE buffer (10 mM Tris-base, 1 mM EDTA, pH 8.0), followed by two more washes in TE buffer and two washes in H_2_O for 10 min each. Finally, the sections were coverslipped with an aqueous mounting medium (Moviol, pH 7).

### Immunostaining

Primary antibodies used were rabbit anti-Calbindin (Swant, dilution 1:2000) and mouse anti-NeuN (Millipore, dilution 1:50). Secondary antibodies were goat anti-mouse Cy3 (Jackson Laboratories) and goat anti-rabbit Alexa488 (Molecular Probes). The sections were rinsed in PBS at 37°C during 5–10 min, followed by a dip in H_2_O at RT. Tissue sections were permeabilized by incubation in 0.1% Triton-X-100 in PBS for 1 h. To prevent unspecific binding of antibodies, the tissue was treated with 20 mM lysine in 0.1 M sodium phosphate (pH 7.4) for 30 min, rinsed twice in PBS for 10 min each, and incubated in blocking buffer (10% FCS in PBS) for 1 h. The incubation with the first antibody diluted in blocking buffer was overnight at 4°C. Sections were washed three times in PBS for 5 min each and blocking solution was added for 45 min-1 h. Incubations with the appropriate secondary antibodies were for 1 h at RT. Sections were washed twice in PBS for 5 min each and treated with DAPI for 5 min. Two more washes in PBS for 5 min were performed before mounting. Sections were coverslipped with an aqueous mounting medium (Moviol with DABCO, pH 8.5).

### Hematoxylin and Eosin (H&E) Staining

The OCT was removed in double-distilled H_2_O (ddH_2_O) for 5–30 min. After that, slices were immerged in Hematoxylin for 6 min and washed in ddH_2_O. Following a dip wash in deionized H_2_O, the differentiation step was performed with 0.1% HCl (in water). The slices were rinsed in deionized H_2_O using constant flux of water from the tap. Sections were checked under the microscope in order to adjust the color (too red: dip in 0.1% NaHCO_3_; too blue: dip in 0.1% HCl).

After incubation in ammonia for 10 min the sections were incubated in 0.1% Eosin for 6 min and rinsed with distilled water. Sections were dehydrated in a graded series of methanol solutions (50%, 80%, 80%, 100%, 100%) followed by two rounds of Xylol and mounting in Eukitt (Fluka).

### Imaging

Sections were viewed and photographed using a BX63 microscope (Olympus) equipped with a digital camera (Olympus DP80) and using the CellSens Dimension software. Using CorelDraw, images were adjusted in contrast and brightness.

## Results

### Calsyntenins Are Expressed in the Developing Neural Tube

**Figure 1:**
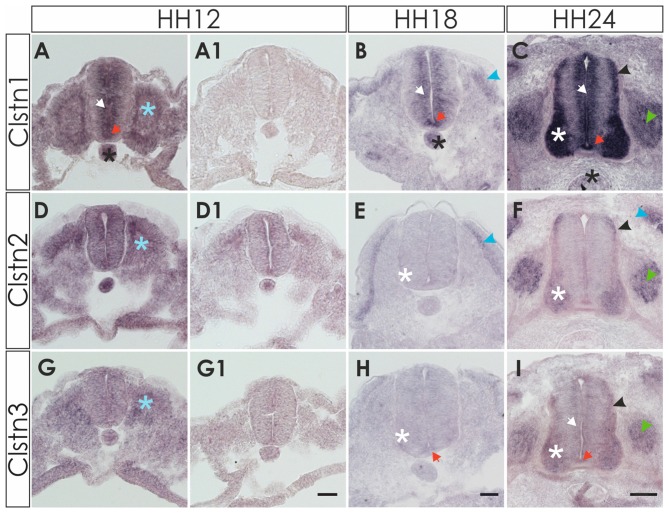
Expression of calsyntenins in early spinal cord development (HH12-HH24). **(A–C)** Calsyntenin-1 (Clstn1) mRNA was found in the ventricular zone (VZ; white arrow) and in the floor plate (orange arrow) already by HH12. Expression was also seen in the notochord (black asterisk) and in somites (blue asterisk; **A**). No staining was detected in an adjacent section processed with the sense probe **(A1)**. At HH18 **(B)**, Clstn1 is still expressed in the VZ (white arrow). The signal in the floor plate was now more distinct (orange arrow). Expression persisted in the notochord (black astersik) and was also observed in the dermamyotome (blue arrowhead). At HH24 **(C)**, Clstn1 mRNA expression is maintained in the VZ (white arrow) and in the floor plate (orange arrow). Highest levels were found in motoneurons (white asterisk). Expression was also found in interneurons all along the dorso-ventral axis (black arrowhead) and in dorsal root ganglia (DRG; green arrowhead). Expression in the notochord decreases with age (black asterisk). **(D–F)** Clstn2 mRNA was found at very low levels, if at all, in the neural tube after its closure. Note that the background in the section processed with the sense probe **(D1)** is relatively high for the youngest stage. At HH12, expression was detected in somites (**D**; blue asterisk). At HH18 **(E)**, expression was more distinct in the dermamyotome (blue arrowhead). In the neural tube, low levels of Clstn2 were only found in very few motoneurons (white asterisk). At HH24 **(F)**, Clstn2 mRNA was found in motoneurons (white asterisk), DRG (green arrowhead) and in interneurons (black arrowhead). Expression persists in the dermamyotome (blue arrowhead). **(G–I)** Clstn3 mRNA is expressed only in somites at HH12 (**G**; blue asterisk; compared with an adjacent section processed with sense probe, **G1**). At HH18 **(H)**, Clstn3 is starting to be expressed at very low levels in motoneurons (white asterisk) and was found in the floor plate (orange arrow). The expression pattern of Clstn3 at HH24 **(I)** is very similar to Clstn1, as mRNA was detected in the VZ (white arrow), motoneurons (white asterisk), interneurons (black arrowhead), DRG (green arrowhead) and the floor plate (orange arrow). Bar 50 μm.

Clstn1 mRNA was detected in the chicken neural tube shortly after its closure, that is at HH12 (E2) at the lumbar level (Figure 1). Between HH12 and HH24, Clstn1 is expressed in the ventricular zone (VZ) and in the floor plate. Motoneurons start to express Clstn1 by HH18 depending on the rostro-caudal level of the spinal cord. As described previously (Alther et al., 2016), Clstn1 is expressed in interneurons, including the dI1 commissural interneurons in the dorsal spinal cord starting at HH22 (not shown; Alther et al., 2016). At HH24, Clstn1 is expressed widely in the spinal cord and in dorsal root ganglia (DRG). Unless stated otherwise, we always compared sections taken from the lumbar spinal cord.

Detection of Clstn2 was more difficult, as expression in the neural tube appeared diffuse and very weak at young stages, HH12 to HH18 (Figures 1D,E). By HH24, the expression pattern of Clstn2 is similar to Clstn1, as we found mRNA in motoneurons and interneurons in the spinal cord as well as sensory neurons in the DRG. In contrast to Clstn1, Clstn2 is not expressed in the VZ and is not found in the floor plate.

Clstn3 is expressed at very low levels, if at all, during early spinal cord development (Figures 1G,H). It is found in somites as early as HH12, however. By HH24, its expression pattern resembles the expression pattern of Clstn1 very closely.

All three calsyntenins are expressed in the sclerotome, dermamyotome and neural crest cells, as demonstrated by comparison with markers for somites and dermamyotome (Pax3), as well as neural crest cells (HNK-1), respectively (not shown).

### Calsyntenins Are Differentially Expressed in Motor and Sensory Neurons

**Figure 2:**
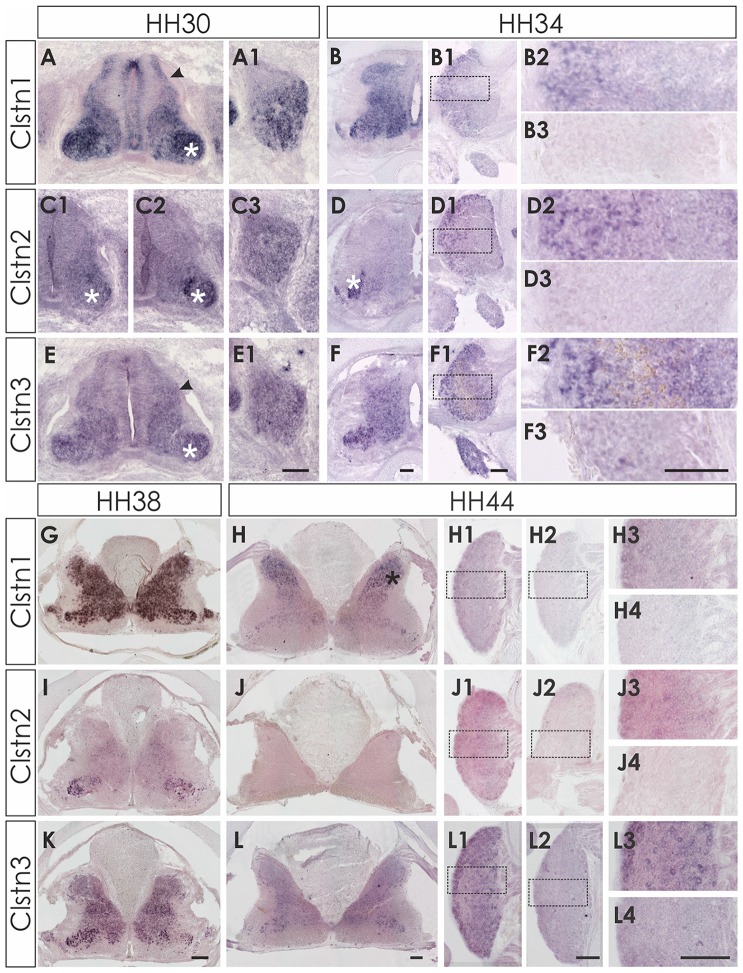
Expression of Clstn1, 2 and 3 mRNA in late spinal cord development (HH30-HH44). The expression of Clstn1 **(A,B,G,H)**, Clstn2 **(C1,C2,D,I,J)** and Clstn3 **(E,F,K,L)** persisted throughout development of the spinal cord. The expression of Clstn1 mRNA at HH30 **(A)** was very similar to HH24. Relative levels changed, as expression levels in motoneurons (white asterisk) were higher than in interneurons (black arrowhead; compare to HH24 in Figure 1). In DRG, Clstn1 mRNA is restricted to the ventro-lateral part **(A1)**. This does not change by HH34 **(B1,B2)**. Expression of Clstn1 in the spinal cord is ubiquitous at HH34 **(B)** and at HH38 **(G)**. By HH44, Clstn1 is expressed predominantly in the dorsal horn (**H**; black asterisk). In DRG, expression is still found in the lateral part **(H1,H3)**. Clstn2 expression in the spinal cord is mainly restricted to motoneurons at HH30 (**C1,C2**; white asterisk). Expression is motoneuron pool-specific and, thus, changed markedly along the rostro-caudal axis (**C2** is more rostral than **C1**). Expression of Clstn2 in DRG is more widespread than Clstn1 at HH30 **(C3)**. With increasing age, the signal is more pronounced in the lateral part **(D1,D2)**. Expression in the spinal cord is still mainly restricted to motoneurons (**D**; white asterisk). This does not change at HH38 **(I)**. However, at HH44 Clstn2 was no longer detected in the spinal cord **(J)**. Similarly, only few cell in the DRG still express Clstn2 at this stage **(J1,J3)**. Clstn3 expression at HH30 **(E)** is similar to HH24 with higher levels of expression in motoneurons (white asterisk) compared to interneurons (black arrowhead). In DRG, Clstn3 is expressed in cells scattered throughout the ganglion at HH30 **(E1)**, HH34 **(F1,F2)** and even at HH44 **(L1,L3)**. In the spinal cord Clstn3 expression was found ubiquitously at HH34 **(F)**, HH38 **(K)** and HH44 **(L)**, although expression levels are higher in the ventral horn. Sections shown in **(B3,D3,F3,H2,H4,J2,J4,L2,L4)** were processed with the respective sense probes. Higher magnification images were taken from the area outlined in **(B1,D1,F1,H1,J1,L1)**. Bar: **(A1–F3)** 100 μm; **(G–L4)** 200 μm.

At E6 (HH29/30) the expression pattern of the three calsyntenins was still comparable to HH24 (Figure 2). Clstn1 is expressed widely in all neurons of the spinal cord as well as in the VZ and in the roof and floor plate (Figure 2A). Expression in the DRG is stronger in the ventro-lateral part than in the dorso-medial part (Figure 2A1). This pattern was more or less maintained at E8/HH34 (Figures 2B,B1,B2) and E12/HH38 (Figure 2G). In the mature spinal cord (HH44/E18), Clstn1 was found predominantly in the dorsal horn (laminae I-III; Figure 2H). Expression levels in motoneurons and sensory neurons have strongly decreased with age and only few neurons maintain Clstn1 expression in the ventral horn and in the DRG, respectively.

Clstn2 expression is more restricted compared to the other family members also in the older spinal cord. In contrast to Clstn1 and Clstn3 (see below), Clstn2 is mainly expressed in motoneurons (Figures 2C,C1,C2). The expression is pool-specific and therefore variable along the rostro-caudal axis (compare Figure 2C1 with 2C2). Expression was also found in the DRG throughout development, with relatively higher levels in the lateral part of the DRG (Figures 2D1,D2). Towards the end of embryonic development, Clstn2 levels decrease even more. By HH44 (Figure 2J) Clstn2 was no longer detected.

The expression pattern of Clstn3 is more similar to Clstn1 than Clstn2 at HH38 and HH44 (Figures 2K,L). At HH44, expression of Clstn1 is stronger in the dorsal horn (Figure 2H), whereas relative Clstn3 levels are higher in the ventral horn (Figure 2L). But both, Clstn1 and Clstn3, in contrast to Clstn2 (Figure 2J), are still found at low levels throughout the gray matter of the spinal cord (Figures 2H,L). In DRG, expression of Clstn3 is less restricted and maintained also in the central part (Figures 2L1,L3).

### Calsyntenins Are Found with Distinct Patterns Throughout the Brain

**Figure 3:**
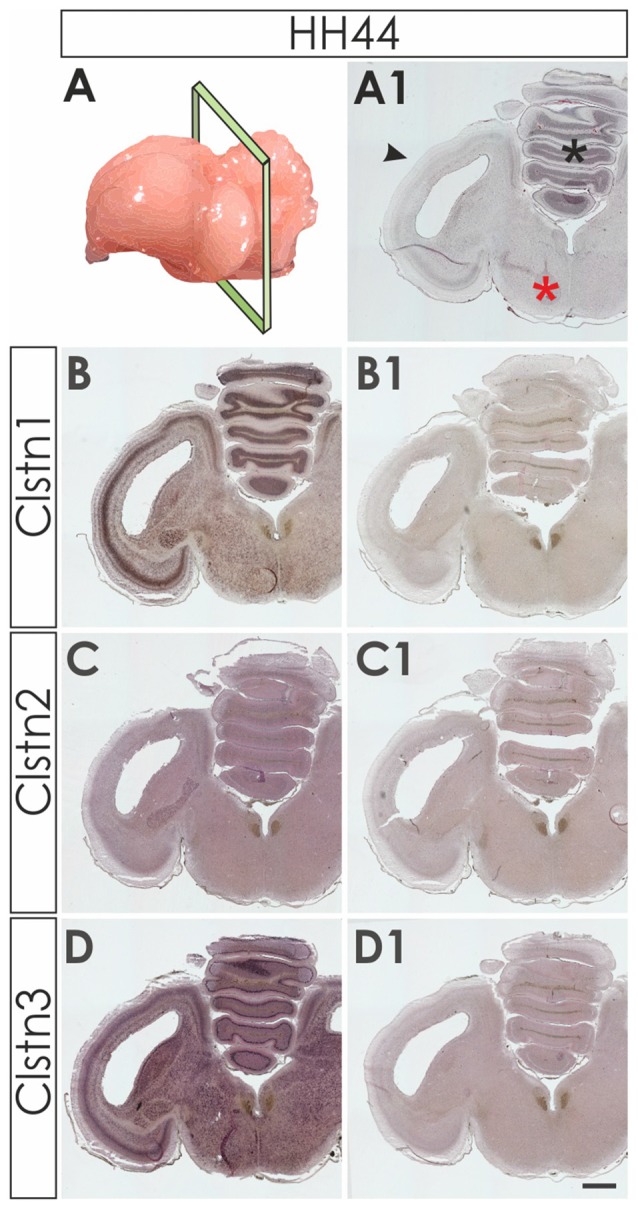
Calsyntenins are widely expressed in the brain shortly before hatching. Coronal sections taken from HH44 brains as indicated in (**A,A1** for H&E stained section) were used to analyze the expression of the calsyntenins. Clstn1 **(B)** and Clstn3 **(D)** but not Clstn2 **(C)** were found in the cerebellum (asterisk in **A1**), the tectum (black arrowhead in **A1**) and in the brain stem (red asterisk in **A1**). Sections **(B1,C1,D1)** were processed with the respective sense probes. Bar: 1 mm.

We first assessed calsyntenin expression patterns in coronal sections of the mature embryonic chicken brain to get an overview of the different brain areas expressing calsyntenin family members (HH44; Figure 3). We found calsyntenins in the cerebellum, the brain stem and the tectum. Their distribution was found to be partially overlapping but each of the three family members had a distinct expression pattern. We thus decided to look at the developing visual system and the cerebellum in more detail. In agreement with our findings from the spinal cord, calsyntenins were not only found in projection neurons but also in their target areas, as demonstrated by the analysis of the visual system, where we detected calsyntenins in the retina and in the tectum.

### Expression of Calsyntenins in the Retino-Tectal System

**Figure 4:**
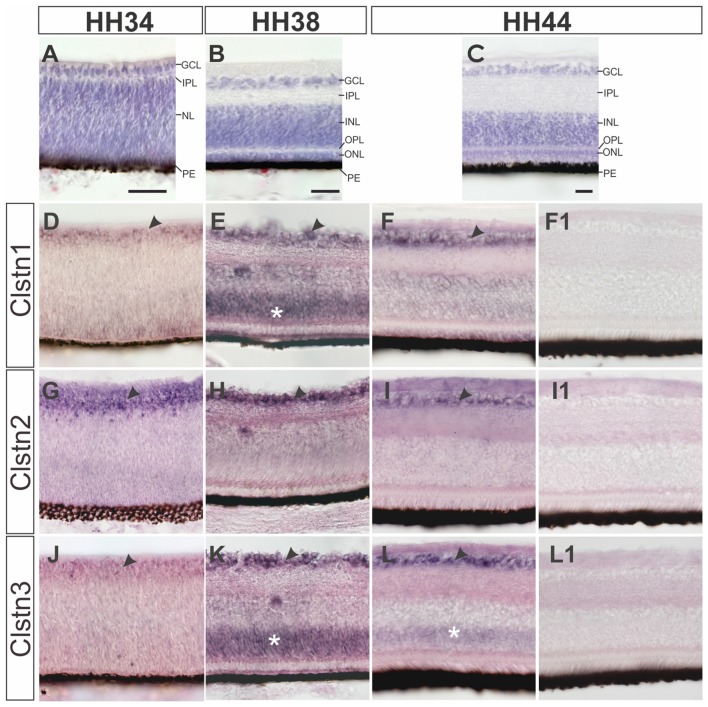
Calsyntenins are expressed in the retina throughout development. The development of the retina is demonstrated by H&E staining at HH34 **(A)**, HH38 **(B)** and HH44 **(C)**. Clstn1 is expressed in retinal ganglion cells (RGCs) throughout development. By HH34 the expression in RGCs is weaker than at older stages (black arrowhead; **D**). Transient expression was found in the outer part of the nuclear layer (white asterisk; HH38, **E**). By HH44, expression levels are low in the inner nuclear layer (INL; but remain high in the RGCs (black arrowhead; **F**). Clstn2 is expressed at high levels only in RGCs (black arrowhead, HH34, **G**; HH38, **H**; HH44, **I**). Clstn3 expression was very similar to Clstn1. Low levels were found in the RGCs at HH34 **(J)**. At HH38 (black arrowhead, **K**) and HH44 **(L)**, expression is high in RGCs. At HH38 **(K)**, Clstn3 mRNA was also found in the outer part of the INL (white asterisk). **(F1,I1,L1)** show sections adjacent to **(F,I,L)** processed with the respective sense probes. Bar: 50 μm.

In contrast to mice, most birds strongly depend on their visual system and therefore its development not only starts early but also reaches maturity before or at the time of hatching (Mey and Thanos, 2000). Layer formation in the retina starts in the second week of development (Figure 4). By HH34 (Figure 4A), the inner plexiform layer (IPL) separates the retinal ganglion cell layer (RGC) from the nuclear layer, containing precursor cells. The IPL contains fibers and synapses between RGCs and amacrine as well as bipolar cells of the inner nuclear layer (INL). By HH38 (Figure 4B), an additional fiber layer, the outer plexiform layer (OPL) has formed and separates the outer nuclear layer containing photoreceptors from the INL. The OPL contains synapses between photoreceptors as well as horizontal and bipolar cells. A functional retina has developed by HH44 (Figure 4C).

All calsyntenins are expressed in RGCs throughout development. Expression in other layers of the retina was found to be transient. For instance, by HH38, Clstn1 and Clstn3 were found in the outer part of the INL (Figures 4E,K). In both cases, expression decreases with increasing age (Figures 4F,L).

**Figure 5:**
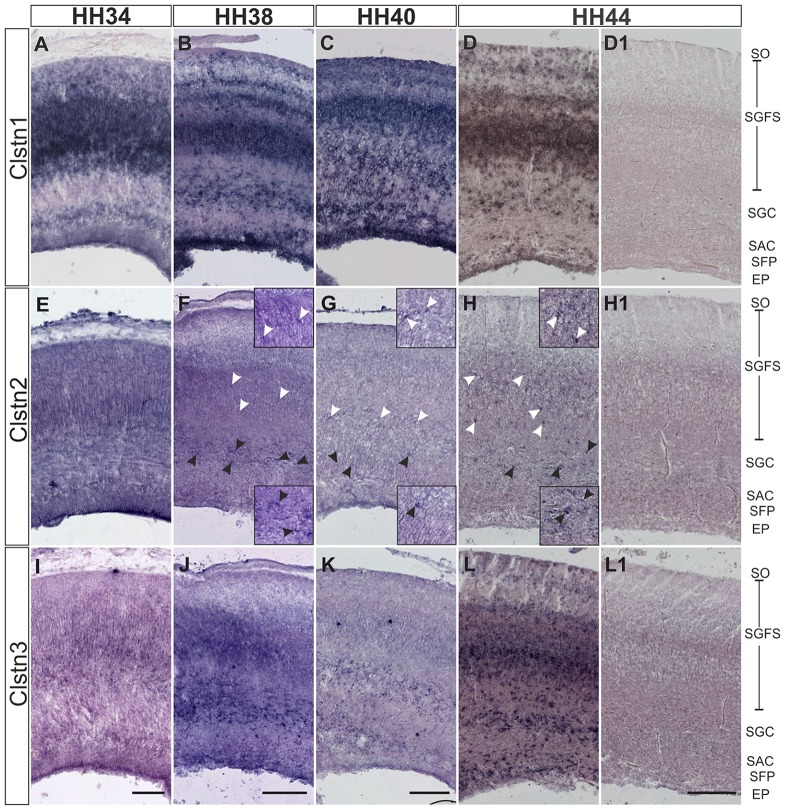
Calsyntenins are expressed in the developing tectum. Clstn1 is expressed in newly forming layers throughout development. By HH34 **(A)** Clstn1 mRNA was found mainly in the upper layers of the developing tectum. As layers form by splitting by HH38 **(B)** and HH40 **(C)**, the Clstn1 expression pattern changes accordingly. By HH44 **(D)**, Clstn1 was found in the SGFS and in the SGC, in some cells of the SAC, as well as in the EP. In contrast, Clstn2 expression is not dynamic. Expression is ubiquitous at HH34 **(E)** but then gradually decreases. It was still readily detectable in most layers by HH38 **(F)** but by HH40 **(G)** and especially by HH44 **(H)** expression of Clstn2 is only retained in very few cells of the SGFS and the SGC. Insets in **(F–H)** are higher magnification views of Clstn2-positive cells in the SGFS (white arrowheads) and in the SGC (black arrowheads). Clstn3 expression resembled Clstn1 at HH34 **(I)**. Expression does not change much by HH38 **(J)**. By HH40 **(K)** expression of Clstn3 has decreased in upper layers. By HH44 **(L)**, expression of Clstn3 is again similar to Clstn1. **(D1,H1,L1)** show sections corresponding to **(D,H,L)** processed with the respective sense probes. SO, stratum opticum; SGFS, stratum griseum and fibrosum superficiale; SGC, stratum griseum centrale; SAC, stratum album centrale; SFP, stratum fibrosum periventriculare; EP, ependymal layer. Bar: 200 μm.

In chicken, the axons of RGCs innervate the superficial layer of the tectum in the second week of embryonic development (Mey and Thanos, 2000). RGC axons can be seen in the superficial layer of the tectum, the stratum opticum (SO) by HH34. At this stage, Clstn1 and Clstn2 are already expressed in the SO (Figures 5A,E). By HH34, Clstn1 is mainly found in the upper (pial) layers of the developing tectum (Figure 5A). In general, Clstn1 expression in the tectum is more dynamic and more prominent than the expression of the other two family members. Clstn1 expression changes with tectal maturation and layer formation. In contrast, levels of Clstn2 are very low and expression appears to be limited to sparse cells of the stratum griseum et fibrosum superficiale (SGFS) and the stratum griseum centrale (SGC; Figures 5E–H). Clstn3 expression is similar to calsyntenin1 at HH34 (Figure 5I), but it stays more or less the same throughout development, as it does not change dynamically with the formation of new layers. Still, by HH44 the two expression patterns are again similar (Figure 5L).

### Calsyntenins Are Expressed Dynamically Throughout Cerebellar Development

**Figure 6:**
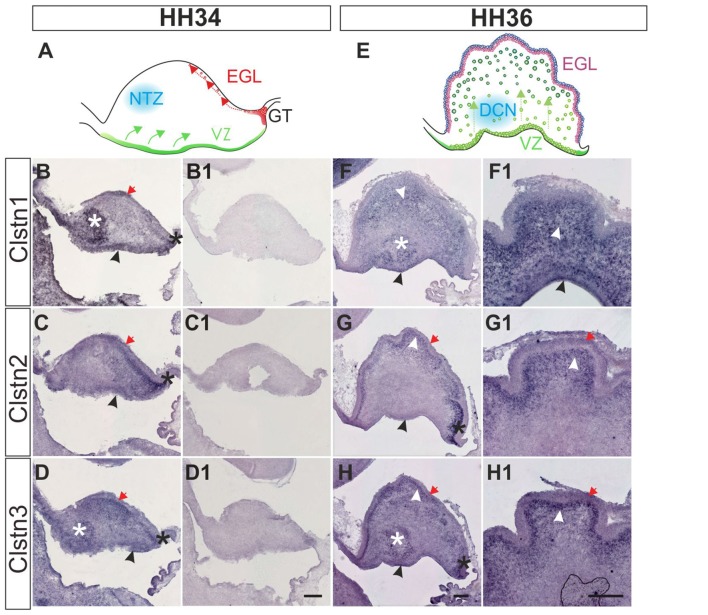
Calsyntenins are expressed in early stages of cerebellar development. Cerebellar development can be analyzed in chicken embryos as early as HH34 **(A)**. In the VZ Purkinje cells are born and migrate away from the ventricle (green). First restricted to the caudal part of the cerebellar anlage, granule cell precursors migrate tangentially to form the external granule cell layer (EGL; red). The blue area outlines cells in the nuclear transitory zone (NTZ; blue; Altman and Bayer, 1997). At this early stage all calsyntenins are expressed in the cerebellar anlage. Clstn1 **(B)** is expressed in the VZ (black arrowhead), the developing EGL (red arrow), in the germinal trigone (black asterisk) and in the NTZ (white asterisk). Clstn2 **(C)** is expressed in the VZ (black arrowhead) and in the developing EGL (red arrow), as well as in the germinal trigone (black asterisk). Expression of Clstn3 **(D)** is similar to Clstn1. It is detected in the VZ (black arrowhead), the developing EGL (red arrow), in the germinal trigone (black asterisk) and in the NTZ (white asterisk). **(B1–D1)** are adjacent sections to those shown in **(B–D)** but processed with the respective sense probes. At HH36 **(E)**, foliation starts because granule cell proliferation in the EGL is rapidly expanding cell numbers. Purkinje cells are still migrating toward the EGL (green). The precursors of the deep cerebellar nuclei (DCN) have descended and are clearly visible in sections taken from the lateral part of the cerebellum. Shown in **(F–H)** are sections taken from the lateral part of a HH36 cerebellum. Those shown in **(F1–H1)** are taken from a medial position of the same cerebellum. At HH36, Clstn1 **(F,F1)** is expressed in ascending Purkinje cells (white arrowhead) and in cells of the DCN (white asterisk), as well as in the VZ (black arrowhead). Clstn2 **(G,G1)** is not found in the central parts of the cerebellar anlage, but high levels of expression are still seen in the germinal trigone (black asterisk) and the granule cell precursors in the EGL (red arrow). It is expressed in Purkinje cells in the developing folds (white arrowhead) and weakly in the VZ (black arrowhead). Clstn3 expression is also similar to Clstn1 at this stage. Clstn3 mRNA is found in the EGL (red arrow), in the germinal trigone (black asterisk), in the VZ (black arrowhead) and in DCN (white asterisk) **(H,H1)**. Bar: 200 μm.

Calsyntenin expression in the cerebellar anlage starts early (Figure 6). The cerebellum has two main germinal zones where neurons are born, the external granule cell layer (EGL) and the VZ (Altman and Bayer, 1997). In addition, the germinal trigone outlines an area at the caudal end of the developing cerebellum which contains stem cells. Granule cells are all born in the EGL but then migrate radially past the developing Purkinje cell layer to form the inner granule cell layer (IGL). The Purkinje cells have the opposite migratory behavior. They are born in the VZ and then migrate towards the pial surface of the cerebellum to form the Purkinje cell layer. Purkinje cells secrete Shh to induce the proliferation of granule cells which in turn induces the foliation of the cerebellum starting around HH36.

At HH34, all three family members are expressed along the VZ, in the external germinal layer, and in the germinal trigone (Figures 6B–D). Expression of Clstn1 is strong in the nuclear transition zone containing precursors of deep cerebellar neurons. Again, similar to what we observed in the spinal cord, retina and tectum, the expression of Clstn3 is more similar to Clstn1 than Clstn2. By HH36, when foliation starts, expression of Clstn1 is seen throughout the cerebellum, except for the proliferating cells in the superficial EGL (Figure 6F). Clstn2 is cleared from the central area of the cerebellar anlage and continues to be expressed in ascending cells and in the EGL (Figure 6G). Unlike Clstn1, it is also found in proliferating granule cell precursors in the outer EGL. Clstn3 distribution resembles Clstn1 in the lateral cerebellum, although in sections taken from the medial cerebellum it looks more like Clstn2 (Figure 6H).

**Figure 7:**
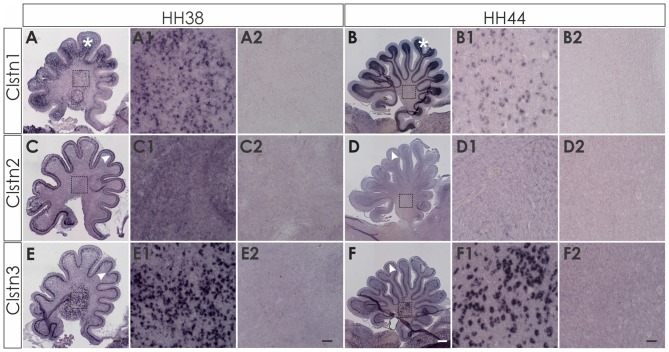
Calsyntenin expression persists throughout cerebellar development. Clstn1 is expressed in the developing inner granule cell layer (IGL; **A**; white asterisk) and persists in the DCN at HH38 **(A,A1)**. This pattern persists to HH44 **(B,B1)**. Clstn2 is expressed in Purkinje cells (white arrowhead) at HH38 **(C)** and still at HH44 **(D)**, although levels are very low by the end of embryonic development (see also Figure 8). Clstn2 was never found in DCN **(C1,D1)**. Clstn3 expression was found in Purkinje cells at HH38 **(E)** and at HH44 **(F)**, as well as in DCN **(E1,F1)**. Sections shown in **(A2–F2)** are equivalent to **(A1–F1)** but were processed with the respective sense probes. **(A1–F2)** represent high-magnification views of the area outlined in **(A–F)**. Bar: **(A,C,E)** 200 μm; **(B,D,F)** 500 μm; **(A1–F2)** 50 μm.

**Figure 8:**
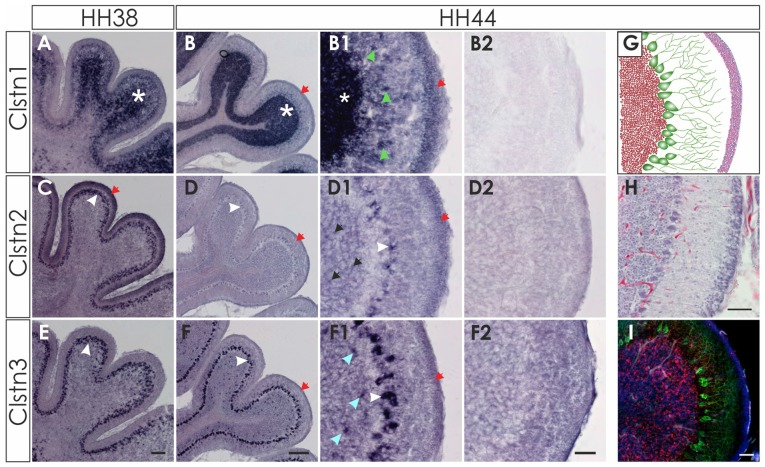
Clstn2 and Clstn3 are mainly expressed in in Purkinje cells. Clstn1 is expressed in the IGL at HH38 (**A**; white asterisk) and at HH44 **(B)**. Because the Clstn1-positive cells differed in size, we concluded that they are not only granule cells but also other cell types. Clstn1-positive interneurons were also found in the molecular layer (ML; **B1**; green arrowhead). Some of the cells in the ML are most likely late migrating granule cells, as Clstn1 mRNA was still found in the EGL at HH44 (red arrow). Clstn2 expression was restricted to Purkinje cells (white arrowhead) at HH38 **(C)** and HH44 **(D)**. At HH44, levels were clearly lower and not all Purkinje cells were positive for Clstn2 mRNA. Some Clstn2-positive cells were also detected in the IGL (black arrow). Clstn3 expression was very similar to Clstn2 at these late stages. Expression was found in Purkinje cells (white arrowhead) at HH38 **(E)** and HH44 **(F)**. Similar to Clstn2, some interneurons in the IGL also expressed Clstn3 (blue arrowhead). **(B2,D2,F2)** Are equivalent sections to those shown in **(B1,D1,F1)**, but processed with the respective sense probes. **(G–I)** By HH44, the cerebellum has almost reached maturity. **(G)** Schematic drawing of granule cells in the IGL (red), Purkinje cells with their dendritic trees (green) and the granule cells in the remaining EGL (pink). **(H)** H&E staining of a sections taken from the same cerebellum as **(B,D,F)**. **(I)** adjacent section to **(H)** stained with antibodies against Calbindin (green) to visualize Purkinje cells, antibodies against NeuN (red) and DAPI (blue). Bar: **(A,C,E)** 100 μm, **(B,D,F)** 200 μm; **(B1–I)** 50 μm.

At later stages of cerebellar development (HH38-HH44; Figure 7), Clstn1 and Clstn3 are maintained in neurons of the deep cerebellar nuclei (DCN). Expression differs in the folds, as Clstn3 is expressed strongly only in Purkinje cells and in some interneurons in the IGL (Figures 7, 8). In contrast, expression of Clstn1 is strongest in granule cells of the IGL. Clstn2 expression decreases with age. Toward the end of embryonic development, expression is maintained in a subset of Purkinje cells (Figures 7, 8). In contrast to the other two family members, Clstn2 is never expressed in neurons of the DCN (Figure 7).

## Discussion

The expression patterns of the three calsyntenins are highly dynamic throughout neural development. They are overlapping but never identical. In general, expression of Clstn1 and Clstn3 were more similar to each other than to Clstn2. Based on the comparative analysis of calsyntenin expression in the adult mouse brain, Clstn1 and Clstn3 were found to be more abundant than Clstn2 (Hintsch et al., 2002). This is in agreement with our results from the developing nervous system. We also found expression of Clstn2 to be more restricted and less variable than the expression of the other two family members. Because detailed analyses of calsyntenin expression patterns are only available for the adult mouse brain (Hintsch et al., 2002), the possibility for comparison of our results from the developing chicken nervous system with mouse is very limited. As described in mouse (Um et al., 2014), we found expression of all calsyntenins in the spinal cord of the chicken embryo (Figures 1, 2). Unfortunately, no information about the cell types expressing calsyntenins in the mouse spinal cord has been published. Expression in Purkinje cells in the adult mouse cerebellum has been reported for Clstn2 and Clstn3 (Hintsch et al., 2002). In accordance with our findings in late stages of cerebellar development, Clstn2 appeared to be expressed in a subset of Purkinje cells, in contrast to Clstn3 that seemed to be found in all Purkinje cells (Figure 8).

In hippocampal cultures, Clstn1 was found to link vesicles to the kinesin motors transporting them along the axons in anterograde direction (Konecna et al., 2006). In addition, a role of Calsyntenin1 in vesicular cargo selection was found in the Golgi apparatus (Ludwig et al., 2009). In line with these functions, calsyntenins are expressed in neurons during neural development, at the time of axonal pathfinding and synaptogenesis. Clstn1 was shown to affect axonal branching of sensory neurons in zebrafish (Ponomareva et al., 2014; Lee et al., 2017). Recently, we characterized a role for Clstn1 in specific trafficking of axon guidance receptors to the growth cone surface in axons that had reached a choice point along their trajectory (Alther et al., 2016). These *in vivo* studies demonstrated that Clstn1 was involved cell-autonomously in the regulation of surface expression of Robo1 receptors on commissural growth cones that was required for axonal exit from the floor-plate area. Despite its dynamic expression in the developing spinal cord, Clstn1’s function in specific delivery of Robo1 to the surface of growth cones in the floor-plate area but not to those of pre-crossing axons appeared to require RabGDI for the temporal regulation. Thus, we concluded that Clstn1 was responsible for cargo selection and linkage of specific vesicles to kinesin motors, whereas additional components were responsible for the exact timing of Calsyntenin function in vesicular trafficking.

A role of all calsyntenins in cargo selection and vesicular trafficking during neural circuit formation is very likely. Although, so far, only Clstn1 has been functionally analyzed during neural circuit formation, the conserved structural features between the family members (Hintsch et al., 2002) and their expression patterns (this study) suggest that also Clstn2 and Clstn3 could contribute to neural development by trafficking selected cargo along axons. Furthermore, non-redundant roles of the calsyntenins in vesicular trafficking are in accordance with findings in synaptogenesis, synaptic plasticity and remodeling (Pettem et al., 2013; Ster et al., 2014; Um et al., 2014; Lipina et al., 2016).

Calsyntenins are not only expressed in growing axons but also in target cells, both intermediate targets, where no synapses are formed, and final targets, where synapses are eventually formed. This is illustrated by the reported expression in the DRG and the spinal cord (Figures 1, 2) but also in the retino-tectal system (Figures 4, 5). It is also consistent with findings during synaptic maturation and function, where dendritic morphology and plasticity of the synapse were shown to depend on Clstn1 (Ster et al., 2014).

The difficulty to analyze the function of individual calsyntenins is due to their structural similarity and their partially overlapping expression patterns, both of which could account for some functional compensation in knockout animals (Pettem et al., 2013; Um et al., 2014). Furthermore, calsyntenins are involved in many steps of neural development which also affects the analysis of their function at later stages or in the adult nervous system. Based on the expression patterns of the three calsyntenins, they could contribute not only to axon growth and guidance as shown by *in vivo* studies (Ponomareva et al., 2014; Alther et al., 2016; Lee et al., 2017) but even prior to this by affecting precursor cell migration and/or differentiation (Figures 1, 6), as we found calsyntenins for instance in the germinal trigone of the developing cerebellum and in the developing EGL (Figure 6). Biochemical and cell biological studies indicate that Clstn1 could affect degeneration of neural function by influencing Aβ levels in patients suffering from Alzheimer’s disease (Steuble et al., 2012; Vagnoni et al., 2012). Taken together, these findings illustrate the importance of calsyntenin function for development and function of the neural networks but they also support the idea that one family of molecules can contribute to neurodevelopmental and neurodegenerative diseases.

Our results are in line with a model that suggests that Calsyntenins are involved in trafficking of specific vesicles during neural development that would allow for the precise localization of surface molecules both in pre- and post-synaptic compartments. The pre- and post-synaptic compartments have to be understood more broadly here, as it is clear that Calsyntenins have a role in neural circuit formation before synaptogenesis onset. This can be concluded from the discovery of Clstn1 as a protein secreted from motoneuron cultures derived from chicken embryos (Vogt et al., 2001) and from functional *in vivo* studies (Ponomareva et al., 2014; Alther et al., 2016). Roles of calsyntenin family members during neural circuit formation prior to synaptogenesis are also suggested by results from the expression study presented here. Although expression of Calsyntenins in general is more restricted during older developmental stages, they are maintained in a cell-type specific manner in line with previously published findings in the adult mouse brain (Hintsch et al., 2002; Um et al., 2014). As demonstrated in axonal navigation in the developing spinal cord (Alther et al., 2016) and as suggested by the only partially overlapping expression patterns, the functions of the Calsyntenin family members are not redundant.

In summary, the expression of calsyntenin family members during neural development suggests a specific and non-redundant function in the regulation of surface molecules on axons and their targets during axon guidance, synaptogenesis and in synaptic plasticity. These functions are in line with reports of genetic and genomic studies implicating calsyntenins in a variety of neurodevelopmental but also neurodegenerative diseases.

## Author Contributions

GRF and TA carried out the experiments. GRF, TA and ETS wrote the manuscript.

## Conflict of Interest Statement

The authors declare that the research was conducted in the absence of any commercial or financial relationships that could be construed as a potential conflict of interest.
